# Metabolic clues to aging: exploring the role of circulating metabolites in frailty, sarcopenia and vascular aging related traits and diseases

**DOI:** 10.3389/fgene.2024.1353908

**Published:** 2024-02-13

**Authors:** Zonghao Qian, Yuzhen Huang, Yucong Zhang, Ni Yang, Ziwei Fang, Cuntai Zhang, Le Zhang

**Affiliations:** ^1^ Department of Geriatrics, Tongji Hospital, Tongji Medical College, Huazhong University of Science and Technology, Wuhan, China; ^2^ Key Laboratory of Vascular Aging, Ministry of Education, Tongji Hospital, Tongji Medical College, Huazhong University of Science and Technology, Wuhan, China

**Keywords:** mendelian randomization, human blood metabolites, frailty, sarcopenia, vascular aging

## Abstract

**Background:** Physical weakness and cardiovascular risk increase significantly with age, but the underlying biological mechanisms remain largely unknown. This study aims to reveal the causal effect of circulating metabolites on frailty, sarcopenia and vascular aging related traits and diseases through a two-sample Mendelian Randomization (MR) analysis.

**Methods:** Exposures were 486 metabolites analyzed in a genome-wide association study (GWAS), while outcomes included frailty, sarcopenia, arterial stiffness, atherosclerosis, peripheral vascular disease (PAD) and aortic aneurysm. Primary causal estimates were calculated using the inverse-variance weighted (IVW) method. Methods including MR Egger, weighted median, Q-test, and leave-one-out analysis were used for the sensitive analysis.

**Results:** A total of 125 suggestive causative associations between metabolites and outcomes were identified. Seven strong causal links were ultimately identified between six metabolites (kynurenine, pentadecanoate (15:0), 1-arachidonoylglycerophosphocholine, androsterone sulfate, glycine and mannose) and three diseases (sarcopenia, PAD and atherosclerosis). Besides, metabolic pathway analysis identified 13 significant metabolic pathways in 6 age-related diseases. Furthermore, the metabolite-gene interaction networks were constructed.

**Conclusion:** Our research suggested new evidence of the relationship between identified metabolites and 6 age-related diseases, which may hold promise as valuable biomarkers.

## 1 Introduction

The world’s population is living longer, and many countries are witnessing an increase in the number and proportion of older adults in their societies. Projections indicate that by 2030, the global population of people aged 65 and over will exceed one billion (Chen, 2022). This demographic shift poses significant challenges for healthcare systems, particularly in effectively managing the wide range of age-related diseases. It is therefore imperative to prioritize the development of methodologies and conceptual frameworks that can identify the fundamental factors that contribute to age-related diseases, thereby enabling timely intervention.

Aging is one of the most significant contributors to the heightened occurrence of physical disability, along with an augmented susceptibility to diseases (Amarasekera et al., 2021). The elderly are particularly prone to a variety of geriatric syndromes, such as frailty, sarcopenia, and vascular aging related diseases.

Frailty is a clinically recognized condition where older individuals become more vulnerable due to a decline in their physical and functional reserves across various physiological systems, which affects their ability to handle daily or acute stressors (Xue, 2011). The Frailty Index (FI) is used as a measure to assess this condition, taking into account various factors such as disability, diseases, physical and cognitive impairments, psychosocial risks, and geriatric syndromes (Mitnitski et al., 2001). The Rockwood FI, which is based on a deficit accumulation model, is an outcome measure for frailty, which assigns scores of 0 or 1 for each deficit, with the FI being the ratio of the number of deficits to a total of 49 and a higher FI score indicates a more severe level of frailty (Atkins et al., 2021; Chen Z. et al., 2023).

Sarcopenia, a common skeletal muscle syndrome, involves a decrease in muscle mass and function due to aging and is identified by traits like low hand-grip strength, measured using dual-energy X-ray absorptiometry (DEXA) or bioelectrical impedance analysis (BIA) (Cruz-Jentoft and Sayer, 2019; Giovannini et al., 2021). This condition significantly impairs mobility, increases mortality rates and imposes significant healthcare costs on individuals (Zhao et al., 2023).

Vascular aging plays a crucial role in the aging process of organs and systems, exerting a fundamental influence on both physiological and pathological aspects (Zhang and Tao, 2018; Bao et al., 2023; Consortium et al., 2023). Although the elderly population may age without overt disease, various vascular changes may occur, including increased vascular stiffness, which is a representative trait of vascular aging, usually measured using carotid-femoral pulse wave velocity (PWV) (Gómez-Sánchez et al., 2023; Mitchell et al., 2023). The Pulse Wave Arterial Stiffness Index (ASI), indicating arterial stiffness, is calculated using a pulse waveform recorded at the finger (Fung et al., 2019).

Changes in vascular aging can lead to vascular dysfunction, which is of great importance as they often precede clinical manifestations and serve as notable risk factors for diseases associated with vascular aging including atherosclerosis, peripheral vascular disease (PAD) and aortic aneurysm (Zhang and Tao, 2018; Cao et al., 2022). Atherosclerosis, involving the accumulation of fatty material and calcium in arterial walls, leads to artery blockage, and diagnosis of which often involves angiography or catheter-based intravascular ultrasound (Lusis, 2000; Ibañez et al., 2009). PAD, which affects arteries in the limbs and visceral organs, is characterized by leg pain during walking that eases with rest, with the ankle-brachial index (ABI) test being a common diagnostic method (Firnhaber and Powell, 2019). An aortic aneurysm, a ruptured aneurysm in the proximal portion of the descending aorta proceeding from the arch of the aorta, is typically detected using ultrasound (US) and computed tomography angiogram (CTA), with diagnosis focusing on the aorta’s maximum diameter (Moxon et al., 2010).

The circulatory system is a critical conduit for delivering nutrients to tissues and organs. Human blood samples provide a comprehensive record that reflects not only the genetic diversity among individuals but also the variance in their physiological responses. Consistent with this, a growing body of research has underscored the influence of circulating metabolites on the progression of age-related diseases (Kameda et al., 2020; Murthy et al., 2020; Kauko et al., 2021; Lieberg et al., 2021; Paapstel and Kals, 2022; Picca et al., 2022).

The investigation of metabolic biomarkers holds significant potential in elucidating the molecular underpinnings of aging-related diseases, thereby aiding in the development of preventative strategies. For instance, the current diagnostic approach for sarcopenia predominantly relies on sophisticated and equipment-intensive methods, such as computed tomography (CT), to assess muscle mass and function, which is not only costly but also often inaccessible to many patients, suggesting the identification of blood-based biomarkers for the screening and diagnosis of sarcopenia presents a more straightforward and cost-effective alternative (Lian et al., 2023). However, the challenge remains that no biomarker has yet been conclusively identified for the specific diagnosis of conditions such as frailty, sarcopenia, and vascular aging-related traits and diseases. This is partly attributed to the reliance on observational studies, which complicates the establishment of causal relationships due to potential biases arising from confounding factors and reverse causality.

Mendelian randomization (MR), a means of investigating the causal involvement of metabolites in the etiology of human disease, can effectively address numerous biases encountered in observational studies, including confounding factors. In this research, a two-sample MR analysis was conducted, using the latest summary statistics encompassing 486 metabolites (Shin et al., 2014). Our primary objective was to evaluate the causal relationships between these metabolites with frailty, sarcopenia, arterial stiffness, atherosclerosis, peripheral vascular disease (PAD), and aortic aneurysm. In addition, efforts were made to identify common metabolic pathways and related genes that may potentially contribute to the onset and progression of these diseases.

## 2 Materials and methods

### 2.1 Study design

**FIGURE 1:**
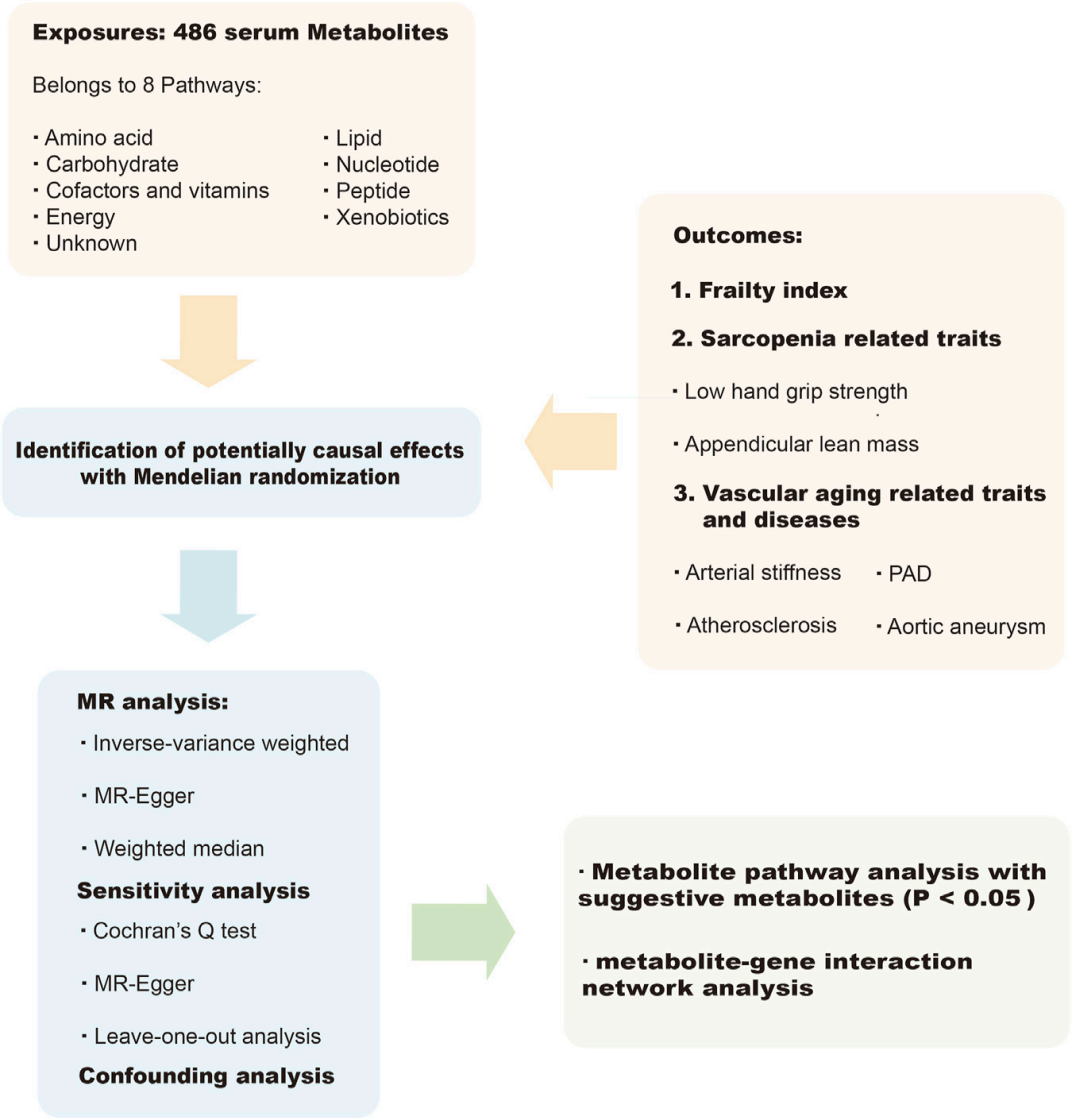
Overview of the current study.

Using a two-sample MR design, we performed a comprehensive evaluation of the causal relationship between circulating metabolites in humans (as the exposure) and the likelihood of frailty, sarcopenia (measured by sarcopenia-related traits, namely, low hand-grip strength and appendicular lean mass (Zhao et al., 2023)), arterial stiffness, atherosclerosis, PAD, and aortic aneurysm (as the outcome). A convincing MR study should adhere to three basic assumptions. Firstly, the genetic instruments must be closely related to the exposure of interest (i.e., metabolites in this study). This ensures that the genetic instruments are relevant to the exposure being studied. Secondly, we ensured that the genetic instruments used in our study were unrelated to the outcome variables (i.e., frailty, sarcopenia, arterial stiffness, atherosclerosis, PAD and aortic aneurysm) and independent of any recognized or unrecognized confounding factors. Thirdly, we assumed that the effects of the genetic instruments on the outcome variables were solely mediated by the exposures of interest, in this case, the circulating metabolites. This assumption helps establish a causal relationship between the exposure and the outcomes. To avoid sample overlap and ensure the robustness of our results, we obtained genetic information from independent genome-wide association study (GWAS) datasets. This approach minimizes the potential for bias and strengthens the validity of our findings. Figure 1 presented an overview of this MR study.

### 2.2 GWAS data for study exposures

We obtained genome-wide association summary datasets for 486 metabolites from the study conducted (Shin et al., 2014). This GWAS analysis included data from 7,824 adult individuals from two European cohorts, TwinsUK and KORA, and encompassed approximately 2.1 million SNPs.The complete summary statistics of these datasets can be accessed publicly *via* the Metabolomics GWAS Server (http://metabolomics.helmholtz-muenchen.de/gwas/). Among the 486 metabolites, a total of 309 were recognized as known metabolites, which could be classified into 8 general metabolic categories according to the Kyoto Encyclopedia of Genes and Genomes (KEGG) database (Kanehisa et al., 2012). These groups include amino acids, carbohydrates, cofactors and vitamins, energy, lipids, nucleotides, peptides, and xenobiotic metabolism. For other 177 metabolites unknown of their chemical characteristic, we excluded them for further analysis.

### 2.3 GWAS data for study outcomes

We used publicly available GWAS summary statistics, the relevant information of the GWAS statistics are presented in Additional file 1: Supplementary Table S1.

Briefly, the genome-wide association study (GWAS) meta-analysis for a frailty index was sourced from Atkins et al.'s study (Atkins et al., 2021). This study encompassed 164,610 UK Biobank participants of European descent, aged between 60 and 70 years at baseline (average age 64.1, standard deviation 2.8), with females constituting 51.3% of the group. It also included 10,616 TwinGene participants of European descent, who were Swedish nationals aged between 41 and 87 years (average age 58.3, standard deviation 7.9), with 5,577 females (52.5%). Additionally, the Swedish Adoption/Twin Study of Aging (SATSA) contributed participants (n = 368) for DNA methylation-related follow-up analyses. These participants were all of European descent, aged between 48 and 93 years (average age 68.6, standard deviation 9.6), and included 223 females (60.6%).

Summary data for low hand-grip strength were derived from a comprehensive meta-analysis GWAS that included 256,523 participants of European ancestry, all aged 60 years or older. This analysis incorporated data from 22 independent cohorts, including the UK Biobank, the US Health and Retirement Study, the Framingham Heart Study, and others. The maximum hand grip strength was recorded, with the low strength cutoff defined as less than 30 kg for males and less than 20 kg for females (Jones et al., 2021). Summary statistics for appendicular lean mass (ALM) were obtained from a GWAS that included 450,243 participants from the UK Biobank Study (Pei et al., 2020).

Genetic variants associated with the arterial stiffness index were extracted from a large-scale GWAS that comprised 127,121 individuals of European ancestry from UK Biobank (Fung et al., 2019).

For atherosclerosis, peripheral artery disease (PAD), and aortic aneurysm, genetic variants were sourced from large, publicly available GWAS databases (https://gwas.mrcieu.ac.uk/). The specific GWAS IDs used for these conditions were finn-b-I9_ATHSCLE_EXNONE for atherosclerosis, finn-b-I9_PAD_EXNONE for PAD, and finn-b-I9_AORTANEUR for aortic aneurysm.

### 2.4 Selection of instrumental variables

Firstly, we extracted SNPs with association thresholds at *p* < 1 × 10^−5^ for each metabolite, which aligned with previous investigations (Cai et al., 2022; Shang et al., 2023). Secondly, SNPs that did not exhibit linkage disequilibrium (LD) with other SNPs (with an r2 value less than 0.01 within a clustering window of 500 kb) were employed as genetic instruments for these metabolites. Thirdly, in order to assess the strength of the chosen SNPs as instruments, we computed the F statistic of instruments for every metabolite and used F statistic of SNP >10 for MR analysis.

### 2.5 MR analysis and sensitivity analysis

The inverse-variance weighted (IVW) method was used as the primary method. We defined them as suggestive features if *p* < 0.05 by IVW. The estimated causal effect of a given metabolite was considered statistically significant if the false discovery rate (FDR) was less than 0.05 or if *p* < 0.05 for all 3 MR analyses (i.e., IVW, weighted median, and MR-Egger). Additionally, several sensitivity analyses were performed. These included: 1) to identify heterogeneity in the association between instrumental variables IVs, we employed the Q-test; 2) we utilized MR-Egger to estimate horizontal pleiotropy 3) we employed additional MR methods including MR Egger and weighted median to enhance the stability and reliability of the findings; 4) individual SNP analysis and leave-one-out analysis were conducted to assess each SNP effect on the overall causal estimate. The R package TwoSampleMR (version 0.5.6) was utilized to run all the analyses in R (version 4.0.0).

### 2.6 Metabolic pathway analysis

The web-based Metaconflict 4.0 (https://www.metaboanalyst.ca/) was utilized to perform a metabolic pathway analysis which includes two datasets: the Small Molecular Pathway database (SMPDB) and the KEGG database. The pathway analysis was considered significant at a threshold of 0.05.

### 2.7 The construction of gene-metabolism-disease network

As fundamental biological processes, metabolism and gene expression work together to maintain homeostasis and to regulate cell growth, survival, and differentiation, which in turn affects the course of disease (Yuan et al., 2013). In order to investigate the relationship between metabolites and aging-related diseases from the transcriptome perspective, we downloaded transcriptome datasets from the Gene Expression Omnibus (GEO) database as shown in Additional file 1: Supplementary Table S6 and the specific information of samples can be found in Additional file 1: Supplementary Table S7. The ‘sva’ package of R was utilized to eliminate batch effects of the datasets. The limma package was used for differential analysis. Genes with *p* < 0.05, |logFC|>0.3 were considered as differentially expressed genes (DEGs). The results were visualized using the ‘gglot2’ package for R. The Kyoto Encyclopedia of Genes and Genomes (KEGG) is a method that is used to find out the relationship between genes and metabolic pathways (Kanehisa et al., 2023). The STITCH software (http://stitch.embl.de/) is a public accessible database of gene-metabolite correlations. The STITCH website was used to build the interaction network between DEGs and significant metabolites, which was visualized with Cytoscape and Adobe illustrator.

### 2.8 Confounding analysis

Despite employing a variety of statistical methods in sensitivity analysis to scrutinize potential violations of MR assumptions, we extended our scrutiny to the Phenoscanner V2 website (http://www.phenoscanner.medchsl.cam.ac.uk/). Our aim was to investigate whether the single nucleotide polymorphisms (SNPs) associated with identified metabolites were simultaneously linked to common risk factors. Such associations could introduce biases in the MR estimates. If the SNPs exhibited associations with these potential confounders, reaching a significance threshold of *p* < 1 × 10^−5^, we proceeded to replicate the inverse-variance weighted (IVW) analysis after excluding these SNPs. This additional step was undertaken to validate and ensure the robustness of our results.

For sarcopenia, for independence assumption, we considered osteoporosis as major confounders (Liu et al., 2022; Ma et al., 2022). We therefore carried out a sensitivity analysis with additional exclusion of SNPs showing genome-wide significant associations with osteoporosis by manually searching the Phenoscanner V2 website.

For atherosclerosis and PAD, for independence assumption, we considered type 2 diabetes mellitus (T2DM), BMI, smoking hypertension, inflammation factors (CRP and IL-6), LDL, HDL, or apolipoprotein as major confounders (Liu et al., 2023). We therefore carried out a sensitivity analysis with additional exclusion of SNPs by manually searching the Phenoscanner V2 website.

## 3 Results

### 3.1 Selection of IVs

The number of selected IVs for 486 metabolites ranged from 3 to 485, with a median number of 15. All F-values for inclusion of SNPs>10.

### 3.2 Causal effects of metabolites on frailty, sarcopenia, vascular age-related traits and diseases

**FIGURE 2:**
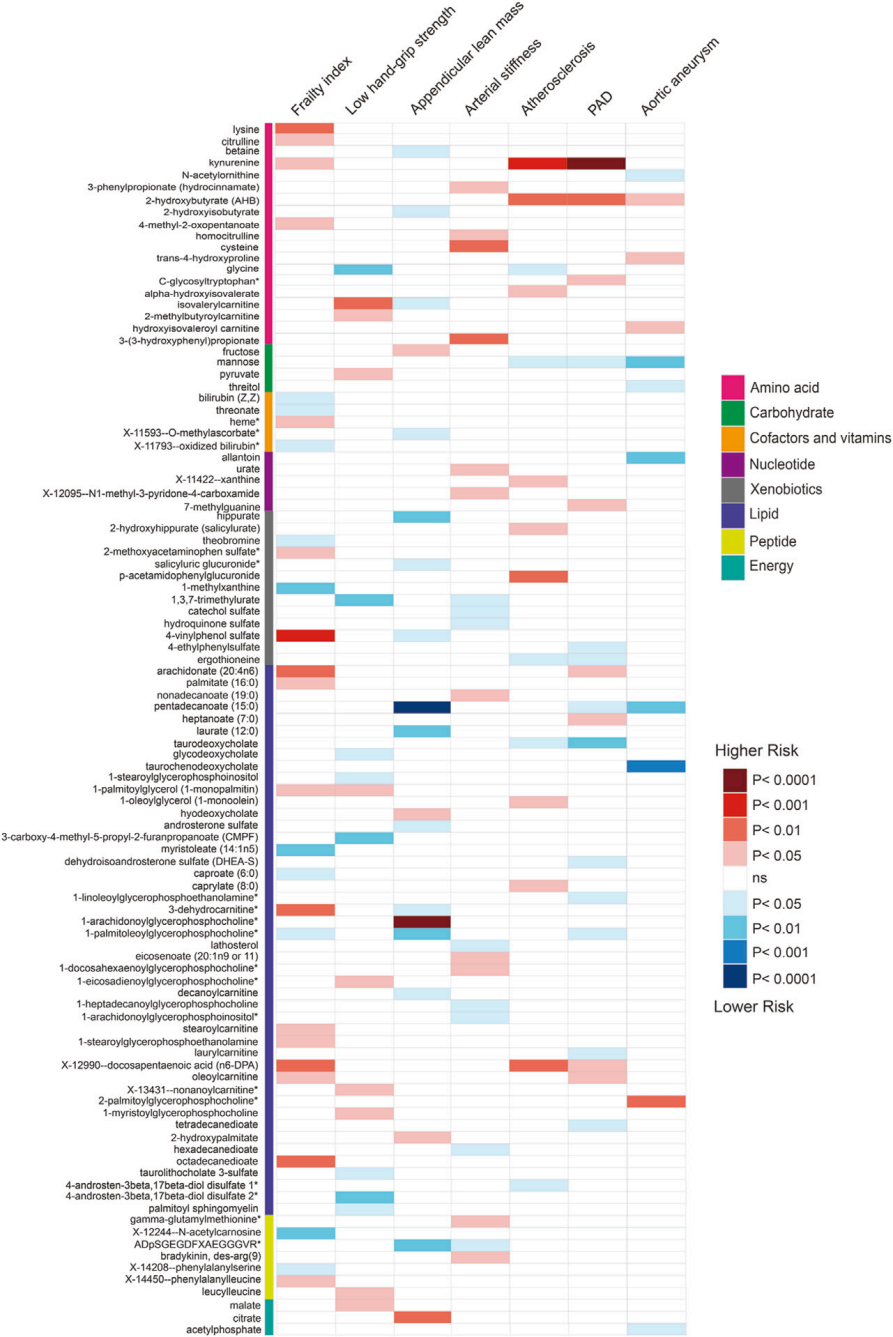
Associations of suggestive metabolites on the risk of the frailty, sarcopenia, arterial stiffness, atherosclerosis, PAD and aortic aneurysm based on MR by IVW.

**FIGURE 3:**
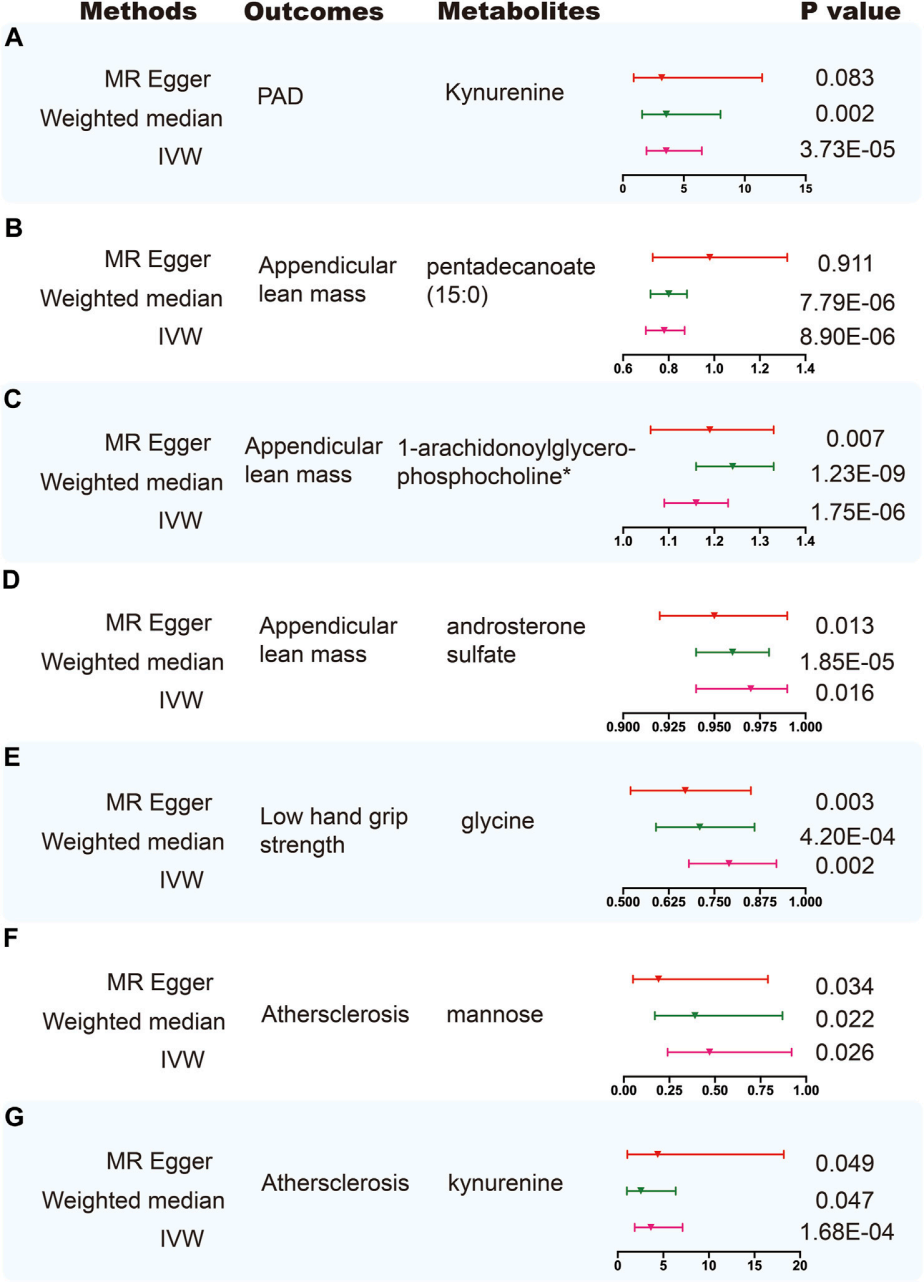
Sensitivity analysis for significant metabolites on outcome phenotypes. **(A)** Sensitivity analysis for the significant MR association between PAD and kynurenine. **(B)** Sensitivity analysis for the significant MR association between appendicular lean mass and pentadecanoate (15:0). **(C)** Sensitivity analysis for the significant MR association between appendicular lean mass and 1-arachidonoylglycerophosphocholine*. **(D)** Sensitivity analysis for the significant MR association between appendicular lean mass and androsterone sulfate. **(E)** Sensitivity analysis for the significant MR association between low hand grip strength and glycine. **(F)** Sensitivity analysis for the significant MR association between atherosclerosis and mannose. **(G)** Sensitivity analysis for the significant MR association between atherosclerosis and kynurenine.

Using selected instrumental variables, we excluded 177 metabolites with unknown characteristics and assessed the causal association between 486 metabolites and frailty, sarcopenia, arterial stiffness, atherosclerosis, PAD and aortic aneurysm. Through our analysis, a total of 125 suggestive associations (*p* < 0.05) were identified (Figure 2). By employing the IVW method and correcting for multiple testing, we observed three causal associations that reached statistical significance (FDR<0.05). They were as follows: PAD, kynurenine (odds ratio [OR] = 3.55, 95% confidence intervals CI 1.94–6.48, FDR<0.05, *p* = 3.73E-05); appendicular lean mass, pentadecanoate (15:0) (odds ratio [OR] = 0.78, 95% confidence intervals CI 0.7–0.87, FDR<0.01, *p* = 8.90E-06); appendicular lean mass, 1-arachidonoylglycerophosphocholine* (odds ratio [OR] = 1.16, 95% confidence intervals CI 1.09–1.23, FDR<0.001, *p* = 1.75E-06). Besides, we found five associations that passed all sensitivity analyses (*p* < 0.05) and the direction of these genetically predicted serum metabolites was consistent across 3 MR methods as shown in Figure 3. They were as follows: appendicular lean mass, 1-arachidonoylglycerophosphocholine*(P_IVW_ = 1.75E-06, P_weighted meidan_ = 1.23E-09, P_MR Egger_ = 0.007); appendicular lean mass, androsterone sulfate (P_IVW_ = 0.016, P_weighted meidan_ = 1.85E-05, P_MR Egger_ = 0.013); low hand grip strength, glycine (P_IVW_ = 0.002, P_weighted meidan_ = 4.20E-04, P_MR Egger_ = 0.003); atherosclerosis, mannose (P_IVW_ = 0.026, P_weighted meidan_ = 0.022, P_MR Egger_ = 0.034); atherosclerosis, kynurenine (P_IVW_ = 1.68E-04, P_weighted meidan_ = 0.047, P_MR Egger_ = 0.049).

### 3.3 Sensitivity analysis

**FIGURE 4:**
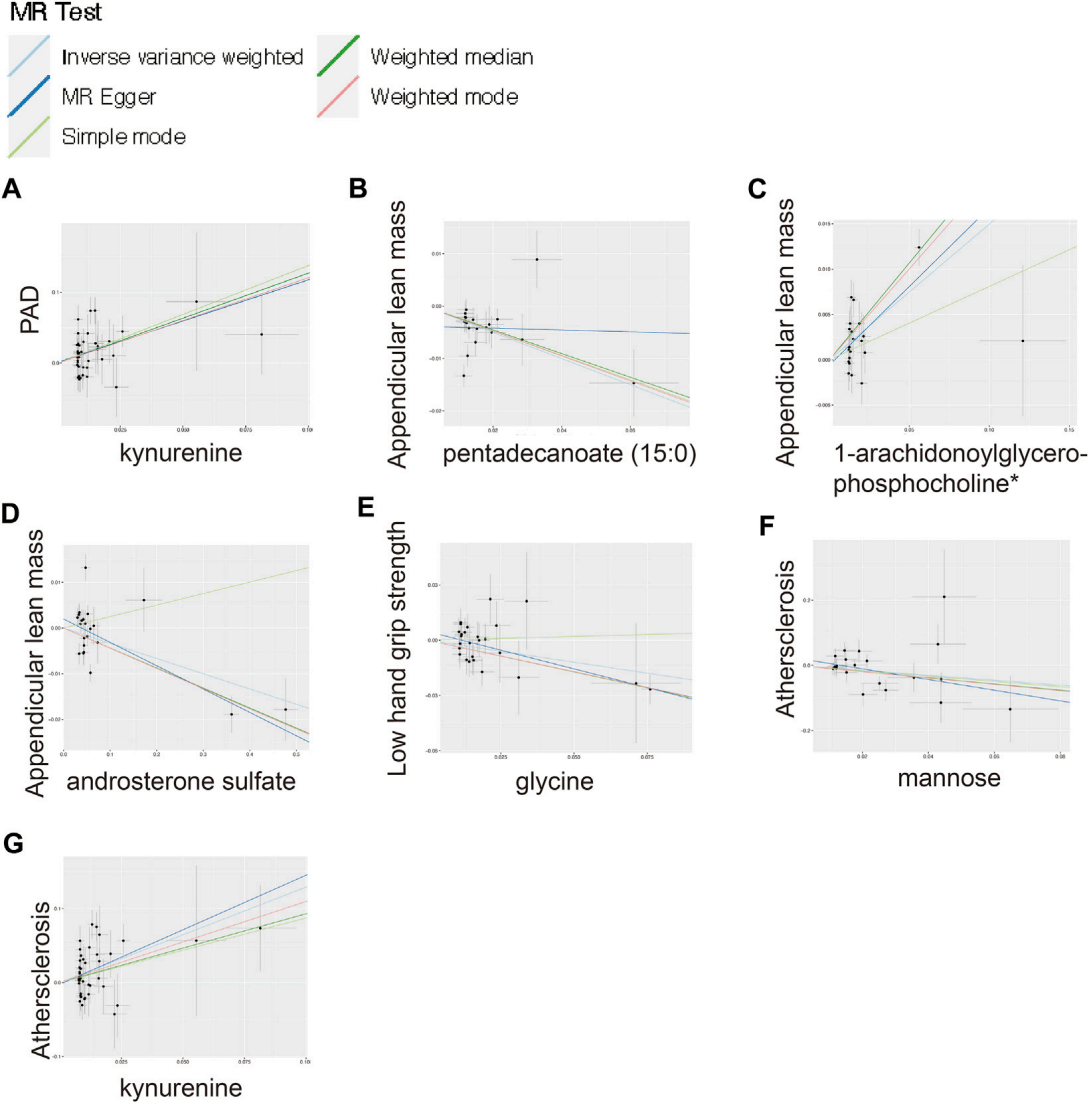
Scatter plot for the significant MR associations. **(A)** Scatter plot for the significant MR association between kynurenine and PAD. **(B)** Scatter plot for the significant MR association between appendicular lean mass and pentadecanoate (15:0). **(C)** Scatter plot for the significant MR association between appendicular lean mass and 1-arachidonoylglycerophosphocholine*. **(D)** Scatter plot for the significant MR association between appendicular lean mass and androsterone sulfate. **(E)** Scatter plot for the significant MR association between low hand grip strength and glycine. **(F)** Scatter plot for the significant MR association between atherosclerosis and mannose. **(G)** Scatter plot for the significant MR association between atherosclerosis and kynurenine.

**FIGURE 5:**
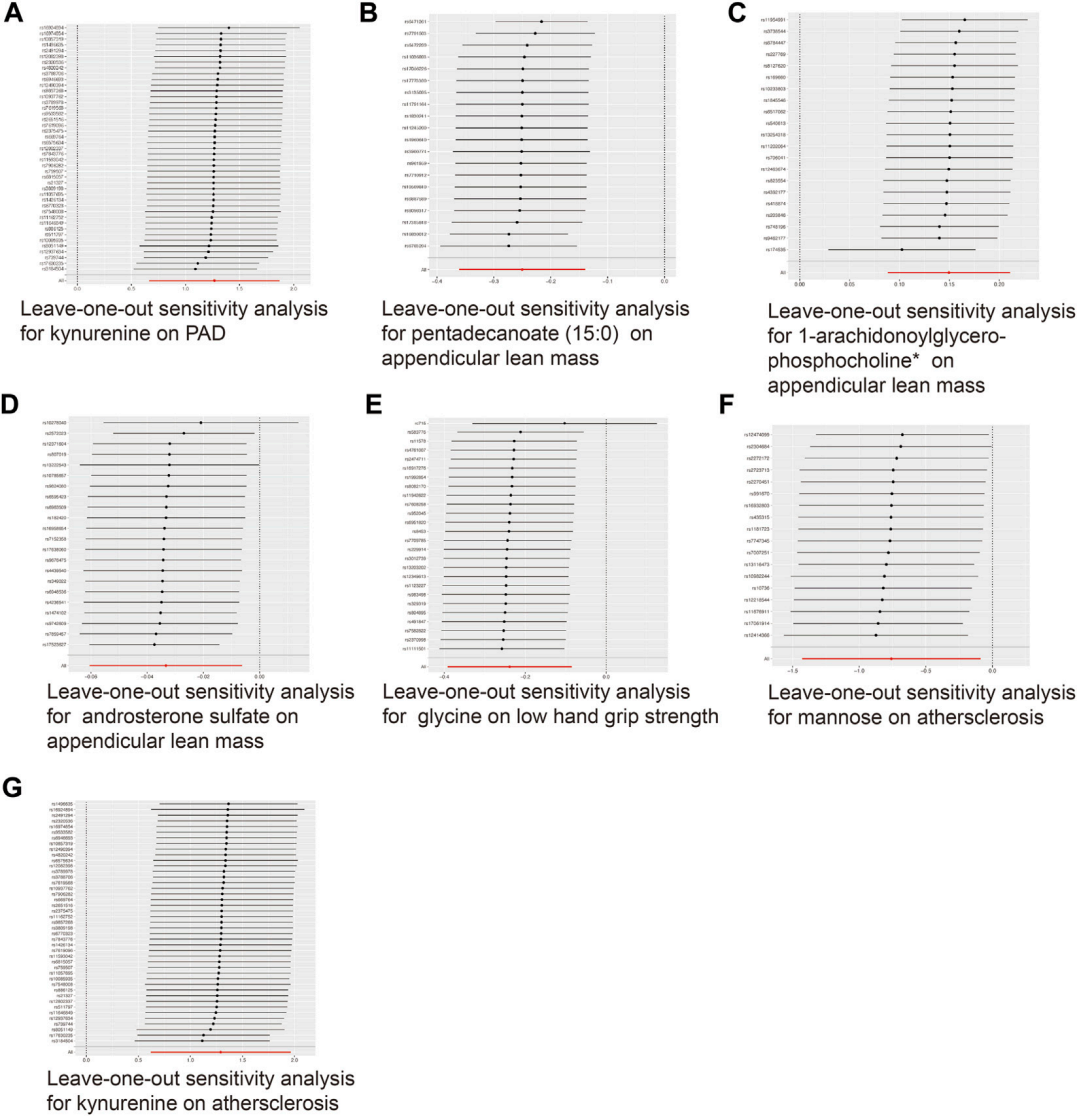
Leave-one-out analysis for the significant MR associations. **(A)** Leave-one-out analysis for the significant MR association between kynurenine and PAD. **(B)** Leave-one-out analysis for the significant MR association between appendicular lean mass and pentadecanoate (15:0). **(C)** Leave-one-out analysis for the significant MR association between appendicular lean mass and 1-arachidonoylglycerophosphocholine*. **(D)** Leave-one-out analysis for the significant MR association between appendicular lean mass and androsterone sulfate. **(E)** Leave-one-out analysis for the significant MR association between low hand grip strength and glycine. **(F)** Leave-one-out analysis for the significant MR association between atherosclerosis and mannose. **(G)** Leave-one-out analysis for the significant MR association between atherosclerosis and kynurenine.

To ensure the accuracy of the MR estimate and avoid any potential horizontal pleiotropy, sensitivity analyses were conducted. The results of these analyses for significant causal associations are presented in Figures 3–5. Leave-one-out analyses for the three metabolites indicated that the estimates were not biased by a single SNP, as shown in Figure 5, suggesting that the estimates remained valid. For a comprehensive overview of the sensitive and pleiotropy analyses, refer to Additional file 1: Supplementary Table S4.

### 3.4 Metabolic pathway analysis of differential serum metabolites and genes in diseases

**FIGURE 6:**
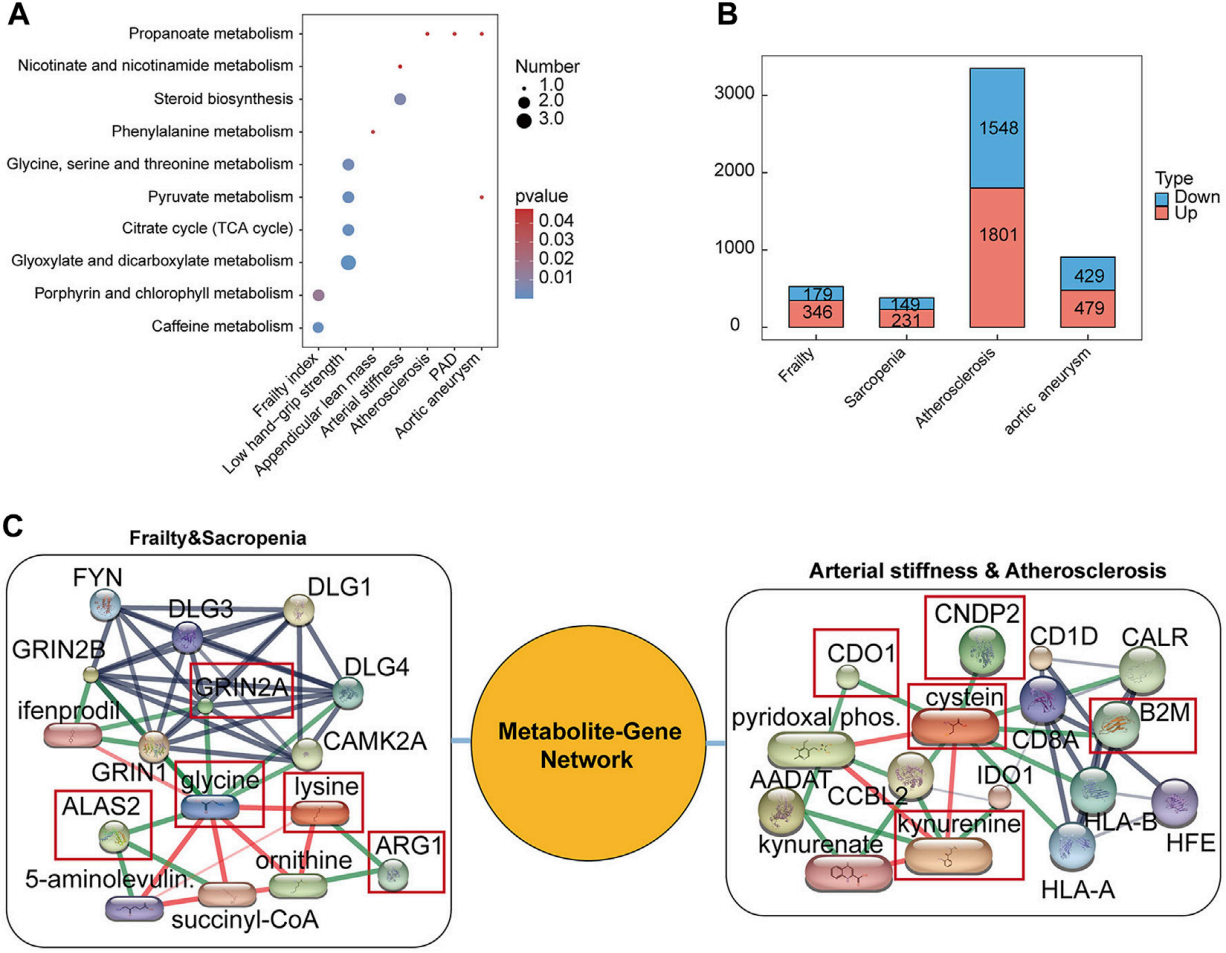
Metabolic pathway analysis of differential serum metabolites and genes in diseases. **(A)** The metabolic pathways of differential metabolites in diseases. The number size represents the amount of metabolites enriched in the metabolic pathway. **(B)** Differential expression gene numbers. **(C)** The network of metabolite-gene-.disease.

Next, we carried out the metabolic pathway enrichment of the differential serum metabolites for all diseases and results as shown in Figure 6A and Additional file 1: Supplementary Table S5. A total of 13 significant metabolic pathways were identified through metabolic pathway analysis. Our results show that “Caffeine metabolism” (*p* = 0.0016409), “Porphyrin and chlorophyll metabolism” (*p* = 0.014803) pathways were found to be associated with the pathogenetic process of frailty; “Phenylalanine metabolism” (*p* = 0.038151) pathway was found to be associated with the pathogenetic process of appendicular lean mass; “Glyoxylate and dicarboxylate metabolism” (*p* = 0.0000778), “Citrate cycle (TCA cycle)” (*p* = 0.0015462), “Pyruvate metabolism” (*p* = 0.001875), “Glycine, serine and threonine metabolism” (*p* = 0.0042247) pathways were found to be associated with the pathogenetic process of low hand-grip strength; “Steroid biosynthesis” (*p* = 0.0068085), “Nicotinate and nicotinamide metabolism” (*p* = 0.04752) pathways were found to be associated with the pathogenetic process of arterial stiffness; “Propanoate metabolism” (*p* = 0.043887) was found to be associated with the pathogenetic process of atherosclerosis; “Propanoate metabolism” (*p* = 0.043887) pathway was also found to be associated with the pathogenetic process of PAD; “Pyruvate metabolism” (*p* = 0.042006), “Propanoate metabolism” (*p* = 0.043887) pathways were found to be associated with the pathogenetic process of aortic aneurysm.

Moreover, to explore the correlation among metabolites-genes-diseases, we had downloaded the transcriptomic data of age-related diseases in GEO database and screened the disease-related differential genes according to |logFC|>0.3, *p* < 0.05. Finally we built the network of metabolites-genes-diseases using the STITCH database. The results are shown in Figure 6B and Additional file 1: Supplementary Table S8.

### 3.5 The metabolite-gene-disease network

Here, we draw a map of the metabolite-gene-disease network in Figure 6C and Additional file 1: Supplementary Table S9. In frailty and sarcopenia, up-DEGs including GRN2A (combined score: 0.991) and down-DEG ALAS2 (combined score: 0.991) interacted with glycine. The up-DEG ARG1 (combined score: 0.994) interacted with lysine. In addition, there was an interaction between two metabolites, glycine and lysine (combined score: 0.991). While, in atherosclerosis, interactions were found between up-DEGs including B2M (combined score: 0.978), CNDP2 (combined score: 0.969) interacted with cysteine. CDO1 is a down-DEG, and an interaction existed between CDO1 and cysteine (combined score: 0.986). There is also an interaction between both metabolites of kynurenine and cysteine (combined score: 0.909).

### 3.6 Confounding analysis

For the identified candidate metabolites (kynurenine, pentadecanoate (15:0), 1-arachidonoylglycerophosphocholine*, androsterone sulfate, glycine, mannose), we further performed a manual investigation of metabolism-related SNPs for the second most common traits (osteoporosis for sarcopenia, while T2DM, BMI, smoking hypertension, CRP, IL-6, LDL, HDL for atherosclerosis and PAD). Through a thorough examination using the Phenoscanner, we found that the SNPs related to the identified metabolites were not associated with any confounding factors.

## 4 Discussion

In this study, we successfully identified a total of 125 causal connections between frailty, sarcopenia, arterial stiffness, atherosclerosis, PAD and aortic aneurysm, and 103 metabolites. Among these metabolites, seven showed significant and robust associations with these age-related diseases and traits after multiple-testing correction and all sensitivity analyses. Furthermore, an examination of six age-related diseases and traits revealed the involvement of thirteen significant pathways. In addition, a comprehensive network analysis of metabolites, genes and diseases was performed, providing new insights into understanding the impact of gene-environment interactions on the development of age-related diseases and offering potential ideas for more targeted therapies.

As lifespan expectancy increases, the prevention and treatment of age-related diseases is emerging as a major global public health concern for the coming century (Beard et al., 2016). Elderly are most likely to die prematurely from frailty, sarcopenia, and cardiovascular diseases (CVD) (Dent et al., 2016; Beaudart et al., 2017; Townsend et al., 2022). Furthermore, these conditions are interdependent, as patients who have frailty or sarcopenia in addition to CVD will have a worse prognosis than those who have CVD alone (Sergi et al., 2015; Shinmura, 2016; Damluji et al., 2023). Early detection of these conditions has the potential to significantly improve the quality of life of the older people affected (Cruz-Jentoft and Sayer, 2019; Dent et al., 2019). Recent research indicates a strong correlation between the development and progression of age-related diseases and age-related metabolic changes (Ungvari et al., 2020; Covarrubias et al., 2021; Sudar-Milovanovic et al., 2022). Serum metabolites are currently considered to be one of the most promising biological markers for the early detection of age-related diseases (Curcio et al., 2016; Pujos-Guillot et al., 2018). Therefore, it is of great value to investigate the relationship between metabolites and diseases.

In the frailty research, a plethora of potential blood biomarkers has been proposed, spanning inflammation markers such as C-reactive protein (CRP), interleukin-6, TNF-α, ICAM-1, MCP-1, TNFR2 (Liu et al., 2016; Tran Van Hoi et al., 2023), growth factors exemplified by growth differentiation factor 15 (GDF-15) (Arauna et al., 2020), and clinical markers like albumin, creatinine, NT-proBNP (Komici et al., 2020; Mak et al., 2023). Recent strides in metabolomics, employing liquid chromatography-mass spectrometry, have hinted at potential associations between metabolites and frailty (Mak et al., 2023). Depicted in Figure 2, our investigation unveils 27 suggestive associations, as indicated by MR estimates employing IVW. However, to confer the status of frailty biomarkers, these associations necessitate rigorous clinical validation. Within this spectrum, in line with preceding observational studies (Kameda et al., 2020; Pan et al., 2021), our discoveries indicate that genetically predicted acetyl-carnosine and bilirubin may correlate with a diminished risk of frailty, while palmitate aligns with an increased risk.

The demographic affected by sarcopenia often presents a clinical convergence with individuals experiencing frailty, as the symptomatic criteria for diagnosing both conditions bear resemblance, particularly with sarcopenia aligning closely with the subtype of frailty known as physical frailty (Kondoh and Kameda, 2023). A recent systematic review and meta-analysis undertook a comprehensive exploration of presently reported potential blood biomarkers associated with sarcopenia (Lian et al., 2023). Despite the breadth of this review, the incorporated studies exhibited notable divergences due to disparities in research designs, leading to the revelation that the thirteen scrutinized biomarkers held low diagnostic efficacy for screening sarcopenia. This underscores the importance of alternative methodologies, such as Mendelian randomization, which, unencumbered by confounding factors in observational studies, presents a promising avenue for the exploration and validation of novel biomarkers in this specific context (Lian et al., 2023).

Our investigation, guided by Mendelian randomization, has unearthed intriguing revelations about the intricate associations between metabolites and physiological states. One of our noteworthy findings involves pentadecanoate (15:0), or pentadecanoic acid, which we discovered to be significantly linked to diminished appendicular lean mass, thereby posing higher risk for sarcopenia. Pentadecanoic acid (15:0) is known as a biomarker for gauging fat consumption in dairy milk (Risérus and Marklund, 2017). In line with our investigation, a cross-sectional study utilizing serum metabolomics unveiled heightened levels of pentadecanoic acid in individuals afflicted with diabetic sarcopenia (Tan et al., 2023). This long-chain fatty acid, once deposited in muscles, emerges as a factor compromising muscle integrity, impeding protein anabolism, and invoking the cause of lipotoxicity, ultimately triggering a cascade of low-grade inflammation (Chen et al., 2020). Evidence further suggests that the accumulation of pentadecanoic acid (C15:0) in muscles activates apoptosis signaling, contributing to the genesis of sarcopenia (Chen et al., 2020). Thus, pentadecanoic acid emerges as a biomarker with the potential to forecast the onset of sarcopenia.

Our study also uncovered the positive effect of 1-arachidonoylglycerophosphocholine (1-arachidonoyl-GPC) on appendicular lean mass, aligning with recent findings (Sha et al., 2023). Curiously, even though outcomes were derived from disparate participants, a previous Mendelian randomization study cast indications on androsterone sulfate, revealing a negative association with grip strength (Sha et al., 2023). Our investigation echoed this, indicating a negative correlation between androsterone sulfate and appendicular lean mass, adding genetic evidence for the risk of androsterone sulfate in sarcopenia. Yet, these demand further clinical validation and the underlying mechanisms also need to be explored.

Also in line with prior research (Sha et al., 2023), our findings revealed a negative association between glycine and low hand-grip strength. A clinical trial in elderly and young adults showed that supplementing glycine combined with N-acetylcysteine reduced muscle breakdown and enhanced strength (Kumar et al., 2021). Animal studies also demonstrated glycine’s role of counteracting muscle wasting in models of inflammation and cancer cachexia (Ham et al., 2014; Ham et al., 2016). The mechanisms underlying these may the role of glycine in reducing cell damage, oxidative stress, and pro-inflammatory cytokine production, overcoming anabolic resistance to leucine, and enhancing protein synthesis in skeletal muscle cells (Koopman et al., 2017).

As blood vessels age, they become stiffer, a condition known as arterial stiffness, which is a hallmark of vascular aging (Gómez-Sánchez et al., 2023; Mitchell et al., 2023). Blood biomarkers that show a correlation with arterial stiffness may enable early detection and monitoring of this condition. Notably, certain biomarkers independently associated with arterial stiffness include inflammatory biomarkers like C-reactive protein (CRP), IL-6, Klotho levels, and aldosterone (Angoff et al., 2021). Our recent findings also demonstrate a positive correlation between circulating Fibulin-1 and brachial-ankle PWV, marking it as an independent risk factor for arterial stiffness (Luo et al., 2022). In our study, we identified 19 metabolites with potential links to arterial stiffness, as suggested by MR estimates of IVW, but these require further clinical validation to confirm their role as biomarkers for arterial stiffness and vascular aging.

Atherosclerosis is a disease primarily driven by aging (Wang et al., 2018). There’s a close link between vascular aging and atherosclerosis, and the vascular remodeling associated with aging increases the likelihood of atherosclerosis (Zhang and Tao, 2018; Ungvari et al., 2020). Previous research has identified circulating metabolites associated with atherosclerosis and its subsequent risks (Jiang et al., 2020; Cao et al., 2023). Our study revealed a negative correlation between mannose and atherosclerosis. Interestingly, a recent observational study indicated that high mannose levels are a characteristic of coronary artery disease with a vulnerable plaque phenotype (Ferrannini et al., 2022). This difference between the previous and present research might be due to variations in research designs, as observational studies may have confounding factors leading to incorrect causal inferences.

Kynurenine (KYN), produced through the breakdown of tryptophan (Trp), is significantly implicated in the aging process (de Bie et al., 2016; Refaey et al., 2017). An animal study showed that high-dose kynurenine diets led to muscle mass loss, while tryptophan supplementation resulted in increased lean mass (Dukes et al., 2015). Elevated kynurenine levels are also linked to increased frailty in humans (Pertovaara et al., 2006; Valdiglesias et al., 2018). Our study also suggests a positive association between kynurenine and frailty. Moreover, cardiovascular diseases are associated with overactivation of the kynurenine pathway (KP) (Ala and Eftekhar, 2022). Clinical evidence showed that a higher KYN/Trp ratio and low Trp levels are strongly associated with advanced atherosclerosis (Gáspár et al., 2021). The KP, regulated by cytokines, plays a role in inflammatory responses in vascular and immune cells, which could be a mechanism linking KP to atherosclerosis (Baumgartner et al., 2019). Our study adds genetic evidence to the positive association between kynurenine and atherosclerosis, as well as peripheral artery disease, a form of atherosclerosis affecting the lower extremities (Bevan and White Solaru, 2020).

Aortic aneurysm is another disease related to vascular aging. Accelerated aneurysm formation and expansion are associated with vascular aging (Chen et al., 2016; Mian et al., 2023). Metabolites play a significant role in the pathogenesis of aortic aneurysms and could serve as new biomarkers for diagnosis. A recent review provided an overview of metabolism in aortic aneurysm biology, including the signaling pathways involved and the significance of metabolites like amino acids (e.g., homocysteine, arginine, tryptophan, taurine), lipids (e.g., polyunsaturated fatty acids), uric acid, and polyphenols (Wang et al., 2023). Our research, using Mendelian randomization, indicates that 11 metabolites have suggestive associations with aortic aneurysm, as indicated by IVW, which might help in identifying new targets for preventing and treating aortic aneurysm.

Our study also uncovered metabolic pathways that may be associated with age-related diseases. Notably, we discovered that sarcopenia and aortic aneurysm share a common metabolic pathway involving pyruvate metabolism. Pyruvate, the end product of glycolysis, is crucial for ATP production in mitochondria, and disruptions in pyruvate metabolism can occur due to mutations in genes responsible for its regulation (Gray et al., 2014). Pyruvate is also known for its anti-aging properties, including potent anti-oxidative stress and anti-inflammatory effects. It helps protect mitochondrial structure, endoplasmic reticulum function, and inhibits cellular apoptosis. Moreover, pyruvate can directly produce NAD^+^(Zhou, 2021). In addition, pyruvate dehydrogenase kinases (PDKs), which regulate pyruvate’s entry into the tricarboxylic acid cycle, are implicated in muscle formation and atrophy, making them potential therapeutic targets for sarcopenia (Kim et al., 2023). An experimental study also highlighted the sarcoprotective role of pyruvate dehydrogenase B (PDHB) (Jiang et al., 2023).

Our findings further indicate that atherosclerosis, peripheral artery disease (PAD), and aortic aneurysm share a common metabolic pathway: propionate metabolism. Propionate, a short-chain fatty acid derived from carbohydrate metabolism during glycolysis, plays a crucial role in cardiovascular health. Studies have shown that short-chain fatty acid metabolism, including propionate metabolism, is significant in cardiovascular diseases (CVD) progression. Specifically, propionate protects the vascular system by first acting on the immune system to inhibit helper T cell activity and then reducing cardiovascular damage caused by hypertension (Zhang et al., 2023). Additionally, propionate mitigates atherosclerosis by modulating intestinal cholesterol metabolism and thereby reducing the inflammatory response in diseased arteries, as demonstrated in studies (Haghikia et al., 2022; Hu et al., 2022). Propionate also reduces abdominal aortic aneurysm through the modulation of regulatory T cells in the colon (Yang et al., 2022). Furthermore, elevated serum levels of propionate are associated with a lower incidence of PAD (Muradi et al., 2022).

Intriguingly, caffeine metabolism has been implicated in frailty. Caffeine is known for its antioxidant, anti-inflammatory, and anti-aging effects, and its beneficial impacts on diseases like CVD (Saraiva et al., 2023). A population-based study revealed a nonlinear association between caffeinated coffee consumption and reduced frailty, especially among non-smokers (Pang et al., 2023). Similarly, a prospective cohort study found that higher caffeine consumption in midlife was linked to a lower likelihood of physical frailty in later life (Chua et al., 2023). However, further research is needed to fully understand the underlying mechanisms of these associations.

Our study identified certain metabolites as potential regulators of genes, which might influence the development of diseases through gene interactions (Ye et al., 2022). We found that glycine was negatively associated with low hand-grip strength, suggesting it could play a role in reducing the risk of sarcopenia. As shown in Figure 6, our research indicates that GRIN2A (NR2A) and ALAS2 may interact with glycine. Consistent with this, previous research reported that GRIN2A is involved in glycine metabolism and encodes ionophilic glutamate receptors (Putnam et al., 2011). However, there’s limited research on the role of GRIN2A in frailty and sarcopenia; our study suggests it might regulate sarcopenia through its interaction with glycine, though further experimental validation is needed.

ALAS2 is crucial for producing large amounts of heme necessary for hemoglobin synthesis (Peoc’h et al., 2019). However, its role in aging-related diseases is not well-studied. Gene transcript results showed that ALAS2 expression levels were associated with strength (Pilling et al., 2016). Furthermore, a study reported that truncating mutations in the ALAS2 gene can lead to elevated glycine levels (Tchaikovskii et al., 2019). The role of ALAS2 in sarcopenia warrants further investigation.

Our research also suggests a positive correlation between lysine and frailty, and an interaction between arginase 1 (ARG1) and lysine. ARG1, which converts arginine to ornithine and urea in the final step of the urea cycle, has been shown to increase in activity and expression with age in muscles (Ballantyne et al., 2015; Pandya et al., 2019). This makes ARG1 a potential therapeutic target for age-related muscle loss (Pandya et al., 2019). Additionally, ARG1-induced nitric oxide deprivation may contribute to vascular aging (Garate-Carrillo et al., 2020; Salminen, 2021).

Cysteine (Cys) is an essential component for the antioxidant function of glutathione (GSH) (Kobayashi et al., 2021). Cys and related aminothiols are markers of oxidative stress and are useful in diagnosing and monitoring conditions like cardiovascular diseases, obesity, and insulin resistance (Pavão et al., 2022). Our study suggests a positive association between cysteine and arterial stiffness. Cysteine dioxygenase type 1 (CDO1) plays a crucial role in amino acid metabolism by converting L-cysteine to cysteine sulfinic acid, thereby reducing cysteine accumulation (Hirschberger et al., 2001; Chen M. et al., 2023). CDO1’s regulation of cysteine metabolism significantly impacts metabolic and neurodegenerative diseases (Perry et al., 1985; Chen M. et al., 2023). Our analysis further revealed a significant association between cysteine and CDO1.

We also found that β2-microglobulin (B2M) interacts with cysteine. B2M is a component of the major histocompatibility complex class I (MHC-I), as stated by Gao et al., 2023 (Gao et al., 2023). Interestingly, B2M levels increase in the blood with aging, and studies involving heterochronic parabiosis identified B2M as a pro-aging factor (Smith et al., 2015). A Clinical study in 2021 have shown that B2M is elevated in elderly people and correlates with oxidative stress biomarkers (Althubiti et al., 2021). Moreover, a cohort study indicated that higher B2M levels were independently associated with greater frailty in older adults (Kim et al., 2017).

In addition, our study revealed an interaction between cysteine and carnosine dipeptidase II (CNDP2). Cytosolic CNDP2 plays a role in maintaining redox homeostasis of cells by recruiting Cys from extracellular GSH, as suggested by Kobayashi et al., 2021 (Kobayashi et al., 2021). Besides, Cndp2 expression was significantly higher in a hypertensive mouse model, and elevated Cndp2 levels may result in reduced carnosine and decreased protection against oxidative stress (Chiu et al., 2014). However, the role of CNDP2 in conditions like frailty, sarcopenia, and vascular aging requires further research in experimental and clinical settings.

There are several limitations to the present study. First, this study was specifically designed to assess the effect of metabolites in blood. Thus, the assessment of metabolite levels in more physiologically relevant tissues, such as muscle tissue, in relation to frailty and sarcopenia, could not be directly investigated within the confines of this specific study. Second, despite the implementation of several strategies to minimize bias, the presence of unmeasured factors that could influence the associations cannot be completely excluded. Third, further experiments, clinical validation and external validation in independent cohorts are essential to confirm the relevance of the identified metabolites as biomarkers and the identified genes’ roles in the age-related diseases.

## Data Availability

The original contributions presented in the study are included in the article/Supplementary Material, further inquiries can be directed to the corresponding authors.
